# Combining Upper Limb Robotic Rehabilitation with Other Therapeutic Approaches after Stroke: Current Status, Rationale, and Challenges

**DOI:** 10.1155/2017/8905637

**Published:** 2017-09-13

**Authors:** Stefano Mazzoleni, Christophe Duret, Anne Gaëlle Grosmaire, Elena Battini

**Affiliations:** ^1^The BioRobotics Institute, Scuola Superiore Sant'Anna, Pisa, Italy; ^2^Rehabilitation Bioengineering Laboratory, Volterra, Italy; ^3^Centre de Rééducation Fonctionnelle Les Trois Soleils, Médecine Physique et de Réadaptation, Unité de Neurorééducation, Boissise-Le-Roi, France; ^4^Centre Hospitalier Sud Francilien, Neurologie, Corbeil-Essonnes, France

## Abstract

A better understanding of the neural substrates that underlie motor recovery after stroke has led to the development of innovative rehabilitation strategies and tools that incorporate key elements of motor skill relearning, that is, intensive motor training involving goal-oriented repeated movements. Robotic devices for the upper limb are increasingly used in rehabilitation. Studies have demonstrated the effectiveness of these devices in reducing motor impairments, but less so for the improvement of upper limb function. Other studies have begun to investigate the benefits of combined approaches that target muscle function (functional electrical stimulation and botulinum toxin injections), modulate neural activity (noninvasive brain stimulation), and enhance motivation (virtual reality) in an attempt to potentialize the benefits of robot-mediated training. The aim of this paper is to overview the current status of such combined treatments and to analyze the rationale behind them.

## 1. Introduction

Significant advances have been made in the management of stroke (including prevention, acute management, and rehabilitation); however cerebrovascular diseases remain the third most common cause of death and the first cause of disability worldwide [1–6]. Stroke causes brain damage, leading to loss of motor function. Upper limb (UL) function is particularly reduced, resulting in disability. Many rehabilitation techniques have been developed over the last decades to facilitate motor recovery of the UL in order to improve functional ability and quality of life [7–10]. They are commonly based on principles of motor skill learning to promote plasticity of motor neural networks. These principles include intensive, repetitive, task-oriented movement-based training [11–19]. A better understanding of the neural substrates of motor relearning has led to the development of innovative strategies and tools to deliver exercise that meets these requirements. Treatments mostly target the neurological impairment (paresis, spasticity, etc.) through the activation of neural circuits or by acting on peripheral effectors. Robotic devices provide exercises that incorporate key elements of motor learning. Advanced robotic systems can offer highly repetitive, reproducible, interactive forms of training for the paretic limb, which are quantifiable. Robotic devices also enable easy and objective assessment of motor performance in standardized conditions by the recording of biomechanical data (i.e., speed, forces) [20–22]. This data can be used to analyze and assess motor recovery in stroke patients [23–26]. Since the 1990s, many other technology-based approaches and innovative pharmaceutical treatments have also been developed for rehabilitation, including virtual reality- (VR-) based systems, botulinum neurotoxin (BoNT) injections, and noninvasive brain stimulation (NIBS) (Direct Current Stimulation (tDCS) and repetitive transcranial magnetic stimulation (rTMS)). There is currently no high-quality evidence to support any of these innovative interventions, despite the fact that some are used in routine practice [27]. By their respective mechanisms of action, each of these treatments could potentiate the effects of robotic therapy, leading to greater improvements in motor capacity. The aim of this paper is to review studies of combined treatments based on robotic rehabilitation and to analyze the rationale behind such approaches.

## 2. Robot-Assisted Upper Limb Rehabilitation after Stroke: Two Decades of Evidence and Misunderstanding

Robotic systems for upper limb rehabilitation have two main designs: (i) exoskeletons, generally based on torque actuators that control each joint of the affected limb to be treated and (ii) end-effectors systems that guide only the most distal part of the affected limb [28–30].

During the last 2 decades, a growing number of robotic devices have been developed (e.g., MIME, ARMin, MIT-MANUS, and NeReBot) to offer intensive training based on repeated movements and challenging task-specific exercises [31]. These devices also provide different forms of sensorimotor feedback to patients, which can positively influence the training outcome [32].

Several systematic reviews and meta-analyses have been carried out on the numerous studies of robotic rehabilitation to assess the effects in patients with stroke with a growing interest in the latest years [32–37].

Despite significant heterogeneity in the types of system evaluated (e.g., distal or proximal UL rehabilitation) as well as the clinical research paradigms used [33], there is a general consensus that robot-assisted upper limb therapy is safe and significantly reduces motor impairment of the limb segments targeted by the robotic device (mainly the shoulder and elbow). However, improvements in motor function, although significant, tend to be small [36]. Moreover, some results suggest that it is the addition of robotic therapy to conventional treatment (CT) that is particularly effective [32, 34] although the effects also depend on the stage at which the therapy is carried out; Bertani et al. [37] and Zhang et al. [32] found that, in patients with chronic stroke, robotic training was more effective in reducing motor impairment than conventional therapy, but not in patients with acute stroke.

Most reviews concluded that robotic therapy does not provide any functional benefit and so does not improve activities of daily living due to the lack of generalization of improvement to untreated joints (especially the wrist and hand). However the latest update of the review conducted by Mehrholz et al. [35] found that, compared to other interventions, robot-assisted arm training may improve activities of daily living in the acute phase after stroke but not in the chronic phase [35]; however they concluded that the quality of evidence was low to very low.

The effect of robotic therapy on muscle tone remains uncertain as only two reviews included this outcome; Bertani et al. [37] found no change while Veerbeek et al. [36] showed a negative effect of robotic therapy on muscle tone.

One raised question is that of the effect of the robotic system itself on improvements in motor outcomes versus simply the provision of highly repetitive treatment. Current evidence suggests it is the large number of repetitions that is effective since there are no differences in outcome between robot-assisted therapy and dose-matched conventional therapy [34]. In the future, robotic systems may become more effective by the use of specific robotic paradigms such as perturbing forces that enhance movement errors. Preliminary studies suggest that such paradigms appear more effective than assistive and simply repetitive practice [38, 39].

Thus two decades after the pioneering study by Aisen et al. [40], a sufficient body of evidence suggests that robot-assisted upper limb rehabilitation improves motor impairment. The effect on muscle overactivity requires further study. It has yet to be established if improvements in function result from generalization of improvements to untrained limb segments. Some reviews suggest that greater functional improvements occur when robotic rehabilitation is carried out in the subacute phase of stroke.

Robotic systems should therefore be considered as vehicles that enable delivery of evidence-based, impairment-oriented treatment, providing highly repetitive, intensive, and interactive treatment that is not possible in usual care. Further improvements in function might come from combining treatments that target different impairments (e.g., weakness and spasticity) and other components of the neural disorder (e.g., interhemispheric imbalance) or are based on a more functional approach (virtual reality, FES).

## 3. Robotic Systems Combined with Other Therapeutic Innovations after Stroke: A General Overview

### 3.1. Functional Electrical Stimulation

The effects of functional electrical stimulation (FES) on upper limb recovery in individuals with stroke have been extensively investigated during the last 30 years. FES activates muscles in a pattern that produces a functionally useful movement [41]. Most FES systems can stimulate up to three specific muscle groups in the upper limb, and studies have shown that this can facilitate recovery of functional reach and grasp movements [42].

The effects of FES on motor function are mixed in the literature. A meta-analysis of 10 randomized controlled trials (RCT) concluded that the addition of FES to conventional therapy did not further improve motor function [43]. In contrast, two systematic reviews found that the addition of FES to a motor training program has a greater effect on improving upper limb functional abilities than training alone or no rehabilitation [44], especially when applied in the subacute phase [45]. The effects may depend on the severity of the impairment. Studies have shown that the addition of FES to usual rehabilitation improves motor function in patients with mild/moderate UL paresis [46] but not severe impairment [47]. Moreover, some studies have suggested that FES is more effective if it is triggered by voluntary muscle contractions (EMG-initiated FES) [48].

These results stimulated the development of hybrid robotic systems combining FES with robotic systems [49]. This is particularly logical since most UL robotic rehabilitation devices only train movements of the shoulder and elbow; thus the association with FES of the hand muscles provides simultaneous, functional training to the whole upper limb. A proof of concept study [50] showed functional improvements on the ARAT scale [51] and the ABILHAND questionnaire [52] following training with a robotic device combined with FES. Another study used FES to facilitate active participation in a virtual reality tracking task by stimulating the deltoid and triceps muscles while the arm was supported by a robot. Although the study was carried out in only 5 patients and was not controlled, the results are encouraging, showing a reduction of motor impairment (Fugl-Meyer score, FM score) [53], improvement in tracking capacity, and a reduction in the need for FES over the course of treatment [54]. A randomized controlled trial in patients with chronic stroke [55] found that a robotic device driven by EMG that provided FES to the wrist was more effective in improving FM scores and manual abilities [51] than robotic therapy alone. More recently, Miyasaka et al. [56] showed that FES of the anterior deltoid and triceps brachii associated with shoulder/elbow robotic training increased range of motion and potentially improved the effectiveness of the robot-assisted rehabilitation. Resquín et al. [57] published a comprehensive description of current hybrid approaches and clinical assessments and concluded that results were promising when the assistance is provided to the distal segments (Wrist/hand) together with the proximal joints.

### 3.2. Repetitive Transcranial Magnetic Stimulation (rTMS)

Transcranial magnetic stimulation (TMS) is a 25-year-old noninvasive technique used to characterize the physiological processes involved in functional consequences of stroke [58]. The use of repetitive transcranial magnetic stimulation (rTMS) for therapeutic purposes is a relatively recent approach. rTMS involves indirect activation of corticospinal cells via a coil placed over the motor cortex, through which a brief high current is passed [59]. It is now well established that low frequency (1 Hz) rTMS has inhibitory effects on the motor cortex and high frequency rTMS has an excitatory effect (5 Hz or more) [60]. Thus, rTMS can be used to treat interhemispheric imbalance [60]. Several studies have suggested that the beneficial effect of rTMS is more marked in subcortical rather than cortical stroke [61, 62].

Along with clinical evaluation, functional magnetic resonance imaging (fMRI), and diffusion tensor imaging, rTMS can be used as a predictor of upper limb motor recovery after stroke [63]. Moreover, it has been shown to predict individuals who are more likely to benefit from robot-based therapy [64] since increases in Box and Block Test (BBT) scores (functional outcome measure) after robotic training have been shown to be correlated with a lower baseline motor evoked potential (MEP) amplitude on rTMS.

A study of six patients with chronic stroke highlighted the need to adapt rTMS to the patient's lesions since rTMS may have different effects on motor reorganization depending on the location of the lesions [65]. A group of European experts found a sufficient body of evidence (level B) to suggest low frequency rTMS of contralesional M1 is effective in improving motor outcomes in patients with chronic stroke [66].

A growing number of studies have investigated the effects of combining various upper limb rehabilitation techniques with rTMS [67–71], with inconclusive results. A recent systematic review with meta-analysis [72] showed that rTMS combined with upper limb training has no additional effect on motor function when compared to upper limb training alone. Hosomi et al. [73] found a modest improvement in patients with subacute stroke following rTMS associated with conventional rehabilitation.

However, despite these mixed results, it seems likely that the association of the relative normalization of cortical excitability by rTMS, with repetitive active robotic training, would potentiate the effects of each treatment, resulting in greater motor recovery [74]. Currently, only a few studies [64] have evaluated the combined effect of rTMS and robotic rehabilitation for the upper limb. However the results are too preliminary to conclude.

### 3.3. Transcranial Direct Current Stimulation (tDCS)

Transcranial Direct Current Stimulation (tDCS) is a noninvasive, painless neural modulation technique. It involves cortical stimulation by a constant and low intensity current delivered via two electrodes placed over the head. Anodal and cathodal tDCS have different effects on the motor cortex. The former has an excitatory effect while the latter inhibits or reduces neuronal activity. Knowledge of these effects is useful as general rule; however numerous other factors also affect inhibition and excitation [75], such as axonal orientation [76]. Several studies have demonstrated that anodal tDCS effectively increases activation of the primary motor cortex [77, 78].

The application of cathodal tDCS over the unaffected motor cortex has been shown to improve the motor control of the proximal upper limb in the case of mild impairment and to worsen control in the case of moderate to severe impairment. It is likely that difference in effect is related to the level of spasticity [79]. Moreover, when used as an adjunct to physical therapy, cathodal tDCS significantly reduces muscle tone and improves activities of daily living [80]. Several studies based on a single-session of cathodal tDCS in patients with chronic stroke showed improved motor performance of the paretic hand [81] and finger movement tasks [82]. However, no evidence was found to support either cathodal tDCS over the contralesional motor cortex or anodal tDCS over the ipsilesional motor cortex [75].

Several studies have investigated the effects of combining tDCS with robot therapy. Edwards et al. [83] showed that raised corticospinal excitability accompanied by reduced cortical inhibition following anodal tDCS persisted during task-specific robotic wrist training, confirming the rational for combining the treatments. However, a randomized trial found no additional effect of tDCS combined with bilateral robotic training in subacute stroke, either with anodal or cathodal tDCS [84]. Equally, Triccas et al. [85] found no further effect of robotic training on upper limb motor function with the association of tDCS. A recent literature analysis found that the addition of tDCS (unilateral anodal or cathodal or bilateral) to robotic training (unilateral or bilateral, distal or proximal) did not result in greater improvements than robotic therapy alone in either the subacute or chronic phase [86].

The timing of the application of tDCS may be important. One study showed that movement smoothness improved only when anodal tDCS was delivered prior to robotic training, rather than during or after robotic training [87]. The phase of stroke may also be important. A recent study demonstrated that bilateral tDCS combined with upper extremity robot-assisted therapy resulted in greater improvements in patients with chronic and subcortical stroke than patients with acute and cortical stroke [88].

It appears that the effects of anodal tDCS (on the affected hemisphere) and cathodal tDCS (on the unaffected hemisphere) combined with robot-assisted arm training are similar. A comparative study in patients with severe impairment found that spasticity and motor function (upper limb section of the Fugl-Meyer Assessment (FMA) scale) improved to a similar extent with both modalities [89].

### 3.4. Virtual Reality

Virtual reality (VR) is an innovative, interactive, and adaptive treatment modality. It can be used to provide sensorimotor training in complex, enriched environments, which would be impossible to design in the real world. VR optimizes patient engagement and can provide mass practice tailored to the individual [90]. VR is considered an important patient-centered tool for stroke rehabilitation because of its characteristics, such as contextualized environments and task-oriented training [91, 92]. Moreover, patients with stroke use similar movement strategies in virtual reality environments as in the natural world [93, 94]. VR can also be used as a research tool to investigate how patients interact with different environments in realistic conditions, allowing a precise evaluation with varying stimuli.

The results of several meta-analyses and systematic reviews suggest that the use of virtual reality as an adjunct to usual care can lead to substantial improvements in upper limb motor function and in activities of daily living when compared with the same dose of conventional care in patients with stroke [95, 96]. A recent review found that VR has positive effects in terms of body function and body structure and that the effects are mainly related to the upper limb. However, the effects on activity and participation are small [97].

Several groups have developed systems that combine VR with robotic devices [98, 99]. The robots used are mostly exoskeletons with passive gravity compensation [100] or assist-as-needed algorithms [101]. The common goal is to optimize engagement in assistive therapy, providing patients with continuous visual feedback on movement quality through kinematic modeling [100]. This technology is novel; therefore no randomized, controlled trials have been carried out; however preliminary studies have shown improvements in motor performance of reaching tasks to visual targets [102] and in manipulation tasks involving a hand-wrist assistive device [103]. Moreover, the latter study also demonstrated increased activation of the sensorimotor cortex during performance of grasp tasks, using functional MRI. Preliminary studies of novel applications combining VR-robots and instrumented gloves [104] suggest that specially designed virtual environments might activate the neural circuits involved in motor skill learning processes by the provision of modified visual feedback. Training finger individuation using a mechatronic-virtual reality system has been demonstrated to be more effective than dose-matched occupational therapy in chronic stroke patients [105] and adaptive robot-mediated training combined with a virtual learning environment has been shown to improve coordination in patients with chronic stroke [106].

### 3.5. Botulinum Toxin

Botulinum neurotoxin (BoNT) is a microbial protein that blocks acetylcholine release at the neuromuscular junction. It is used to reduce dystonia, spasticity, and related disorders. Over the last 2 decades, two BoNT serotypes (A and B) have become widely used in neurorehabilitation. BoNT has been demonstrated to be safe and effective for the treatment of spasticity in adults and children, including upper limb spastic paresis due to stroke and traumatic brain injury (TBI) [107–110].

BoNT injected in upper limb muscles reduces muscle tone, with perceived functional benefits [109]. Some studies have also shown that BoNT treatment is effective in decreasing cocontraction of antagonist muscles, facilitating agonist recruitment and increasing active range of motion [111]. However, improvements in active upper limb function remain to be demonstrated [110]. No improvements have been found on the Barthel Index [112], the Functional Independence Measure (FIM) [113], or measures of quality of life such as the SF-36 [114], particularly in early stages after stroke [115]. This lack of effect of the reduction of spasticity on active function suggests that BoNT injections should be combined with exercise therapy [115].

BoNT and robotic rehabilitation appear to be a natural, synergistic combination since several studies have demonstrated that repetitive movement-based robot therapy can reduce muscle tone as well as motor impairment [116–120]. This combination has been evaluated in a small number of studies in children with cerebral palsy (CP) [121, 122] and in patients with stroke [123–125]. A case study in children with cerebral palsy showed improvements in upper limb coordination and quality of motor performance [121]. Another study compared the effects of upper limb robotic therapy with a combined schedule (same robot-assisted training following BoNT injections) and showed a greater decrease in spasticity in the combined group [124]. Moreover, robotic devices are useful for the evaluation of the effects of BoNT. Kinematic assessment of six children with hemiplegia after BoNT administration using a robotic device showed significant improvements in accuracy and smoothness, which was correlated with the results of clinical scales [122].

## 4. Discussion

Over the last two decades, neurorehabilitation has evolved from being empirically based towards a more evidence-based form of practice, transferring the scientific concepts of neuroplasticity and motor relearning as well as advanced knowledge from clinical studies, into clinical practice. A growing body of evidence has demonstrated that stroke induced-changes in cortical neuronal activity and neural circuits can be influenced by physical interventions that target functional deficits and/or impairments such as weakness and spasticity. A growing number of treatment methods and devices are becoming available to facilitate plastic-reorganization of the central nervous system. However, the optimization of treatment effectiveness might require combined approaches with complementary actions. The following section discusses the rationale for combining robotic therapy with other innovative approaches.

### 4.1. Priming Motor Learning-Based Processes

Motor recovery and/or improvements in motor performance after stroke are driven by motor skill leaning that can be mediated by movement-based rehabilitation paradigms. Robotic rehabilitation systems fully meet the requirements for motor learning, since they facilitate practice-dependent improvements in motor performance that persist over time. Although robust evidence for neurorehabilitation-induced plasticity is lacking, it has been suggested that robot-mediated treatment using adaptive algorithms (assistance or perturbation) might have the potential to enhance neuroplasticity [126].

Noninvasive brain stimulation (NIBS) techniques can transiently modulate brain excitability. An obvious application is to prime motor networks [127], increasing responsiveness to motor learning therapies, particularly by delivering them prior to movement-based training. The association of NIBS and robotic systems has been evaluated in preliminary studies with encouraging results [88, 128]. However, many questions still remain with regard to the optimal use of NIBS alone (e.g., delivery of stimulation to the affected or unaffected hemisphere). These questions should be answered before investigating combined approaches.

One of the aims of combining virtual reality environments with robotic rehabilitation is to enhance patient motivation and engagement in movement-based practice. The association of VR with robot therapy adds a “recreational” component to rehabilitation that distracts patients from fatigue and pain and motivates them to improve their performance [129]. As such, many robots are equipped with user-friendly graphical interfaces to provide games-based exercises. However, to date, engineers have primarily focused on robot control strategies and mechanical issues rather than on interfaces and program content. Thus little attention has been paid to the contribution of the “VR” component of robotic therapy to improvements in performance. The virtual reality interface can provide immediate nonspecific visual and auditory feedback that has been shown to enhance motor performance when added to robotic training [130, 131]. Another interesting potential for VR-robot therapy is the provision of feedback on limb position and on potential movement errors. Grimm and Gharabaghi [100] found that rehabilitation with an exoskeleton combined with a virtual reality-based interface that displays a representation of the user arm can enhance performance on clinical tests as well as improving movement kinematics. This is supported by the fact that skill reacquisition is facilitated by feedback regarding knowledge of performance (attention directed to movement kinematics) more than knowledge of results, in which the goal is to achieve the movement, regardless of the strategy [132]. However, much work remains to be done in this field as evidence for the clinical effectiveness of VR-robotic rehabilitation is lacking.

### 4.2. Facilitating Agonist Muscle Recruitment

Although BoNT injections have been shown to reduce muscle spasticity, studies have failed to find a concomitant improvement in motor function and disability. It is likely that patients “learn” new patterns of movement in order to function as effectively as possible in the presence of spasticity. Once these new patterns are established, they may not change, even if spasticity is reduced. Repetitive, goal-directed movements using robotic devices may change these patterns, facilitating more normal movement and improving functional capacity, particularly since robot-mediated training has been shown to change muscle synergies [133]. However, this remains speculative as evidence is lacking.

FES has obvious applications to facilitate appropriate muscle recruitment, and its use with assist-as needed robotic rehabilitation appears to be an interesting combination. Although studies suggest that robot-mediated therapy should focus more on movement coordination than on muscle activation to be effective [134], EMG driven FES may be appropriate to potentiating active movement, especially in patients with severe impairment. Stimulation of both peripheral effectors and centrally controlled motor planning networks might facilitate motor recovery; however, again, this hypothesis needs to be investigated.

### 4.3. Combining Both Proximal and Distal Training

Currently available robotic devices are mostly single or bijoint-based modules that carry out training of only proximal or distal segments, with a single goal to improve either reaching or grasping ability. Hybrid systems combining FES with robot training can train both proximal and distal segments simultaneously, combining impairment-based therapy with functional-oriented tasks (reach-to-grasp). Although some studies have suggested there are no additional advantages when shoulder/elbow robot-assisted training is combined with functional tasks [135], this approach might need further development from both a technological and a clinical point of view. It is well established that voluntary movements are internally represented as goal-oriented motor actions, rather than movements of segments. It is therefore pertinent to rehabilitate arm and hand function together in a coordinated manner [136].

### 4.4. Optimizing Challenge and Motor Behavior

Engagement in rehabilitation is essential to promote learning [137]. VR and robotic systems require interaction by the patient, enhancing engagement as well as providing a focus on motor tasks. One of the underlying mechanisms is likely the rewards associated with actions; evidence indicates that reward is an effective feedback signal for the regulation of behavior and motor learning [138, 139] and has been shown to enhance motor control after robotic training [140]. VR provides an excellent framework for reward presentation [126]. While robotic control systems mostly use assistive algorithms, the association with a VR games-based program may optimize motor behavior, encouraging the patient to improve his/her own motor performance. However, the specific effects of VR on upper limb robotic training have not been studied.

### 4.5. Robotic Devices for Evaluation: A Complement to Clinical Scales

One advantage of robotic devices is their ability to provide a simple and affordable measurement of motion kinematics in standardized conditions, complementing clinical scales of motor function, with a high resolution and specific metrics [20–22]. Advanced systems can measure and record the kinematics of upper limb trajectories in order to determine indicators of movement performance. Kinematic variables provide a valuable measure of motor performance and movement quality. The utility of robotic devices for evaluation purposes has been demonstrated for the assessment of the effects of BoNT in both children with spastic hemiplegia [122, 141, 142] and patients with stroke [143, 144]. Moreover, kinematic parameters better characterize changes in active motor function after BoNT treatment than clinical scales (e.g., movement velocity and smoothness). However, more work is needed to optimize the use of robot-based measurements in clinical practice, including their use in decision making, and for treatment planning and progression.

## 5. Conclusion

There is currently some evidence to suggest that combination-therapies may be more effective than individual treatment techniques.

This paper highlights the potential benefits of combining upper limb movement-based robotic therapy with other approaches. Much work is, however, required to evaluate and optimize such combination-approaches as current studies have many limitations (small sample sizes, heterogeneity of technological devices and methodological paradigms used).
